# Low-Light Dependence of the Magnetic Field Effect on Cryptochromes: Possible Relevance to Plant Ecology

**DOI:** 10.3389/fpls.2018.00121

**Published:** 2018-02-14

**Authors:** Jacques Vanderstraeten, Philippe Gailly, E. Pascal Malkemper

**Affiliations:** ^1^Environmental and Work Health Research Center, School of Public Health, Université Libre de Bruxelles, Brussels, Belgium; ^2^Institute of Neuroscience, Université Catholique de Louvain, Brussels, Belgium; ^3^Department of General Zoology, Faculty of Biology, University of Duisburg-Essen, Essen, Germany; ^4^Department of Wildlife Management, Faculty of Forestry and Wood Sciences, Czech University of Life Sciences, Praha, Czechia

**Keywords:** *Arabidopsis thaliana*, clock proteins, geomagnetic field, light intensity, magnetoreception, plant growth, static magnetic fields

## Abstract

Various responses to static magnetic fields (MF) have been reported in plants, and it has been suggested that the geomagnetic field influences plant physiology. Accordingly, diverse mechanisms have been proposed to mediate MF effects in plants. The currently most probable sensor candidates are cryptochromes (Cry) which are sensitive to submillitesla MF. Here, we propose a quantitative approach of the MF effect on Cry depending on light intensity, and try to link it to a possible functional role for magnetic sensitivity in plants. Based on a theoretical evaluation and on a review of relevant data on *Arabidopsis thaliana* Cry 1, we point out that the MF effect on the signaling state of Cry, as well as the possible consequences of that effect on certain phenotypes (growth in particular) show parallel dependences on light intensity, being most prominent at low light levels. Based on these findings, we propose that Cry magnetosensitivity in plants could represent an ecological adaptation which regulates the amount of Cry signaling state under low light conditions. That hypothesis would preferentially be tested by studying sensitive and specific endpoints, such as the expression of clock proteins that are downregulated by Cry, but under light intensities lower than those used so far. Finally, we highlight that the low-light dependence of the MF effect described here could also apply to light-dependent functions of animal Cry, in particular magnetoreception which, from the present evaluation, would be based on the magnetic sensitivity of the photoreduction reaction, like in plants.

## Introduction

In plants, diverse responses to static magnetic fields (MF) and to near-null MF have been reported. These responses let suggest that, like the other parameters of the physical environment, the geomagnetic field (GMF) can affect plant physiology (Maffei, 2014; Occhipinti et al., 2014; da Silva and Dobránszki, 2016; Binhi and Prato, 2017). However, no clear biological relevance has emerged so far for a supposed GMF effect in plants. In 2012, it has been shown that the plant photoreceptor proteins cryptochromes (Cry) are sensitive to submillitesla MF (Maeda et al., 2012). In line with that observation, Xu et al. (2012) proposed that the GMF can affect the activity of Cry, based on the report of Cry-dependent responses to near-null MF. Here, we propose a quantitative approach of the MF effect on the signaling state of Cry 1 of the model plant *Arabidopsis thaliana* (*At*Cry 1) depending on light intensity. Recently indeed, the photocycle of *At*Cry 1 has been accurately documented (Procopio et al., 2016), and its magnetic sensitivity has been investigated under conditions of illumination that reflect those of the natural environment (Kattnig et al., 2016). We then propose a reevaluation of the MF effects reported in plants in search of possible convergence with our results and, further, of arguments that would support the hypothesis of a role in plant magnetosensitivity. Finally, on the basis of our evaluation of the light intensity-dependence of the MF effects on Cry, we also briefly discuss the case of animal Cry. Indeed, the Cry proteins have been highly conserved during evolution, from plants to vertebrates (Maeda et al., 2012), and *At*Cry has so far been the usual experimental model for the *in vitro* study of the (presumed) Cry-based magnetoreception in animals (Hore and Mouritsen, 2016).

## The geomagnetic field as part of the plant ecosystem

MF effects on plants have recently been reviewed by Maffei (2014), da Silva and Dobránszki (2016) and Binhi and Prato (2017), with the latter focusing on effects of near-null MF. All authors noted the high heterogeneity of the studies performed and the high variability of the reported results. This heterogeneity concerned both the endpoints (genetic, metabolic, morphological) and the study designs (static vs. time-varying fields, MF intensity). Together with a frequent lack of independent reproducibility, that heterogeneity has so far impeded functional interpretation of plant magnetosensitivity. Still, several hypotheses have been proposed.

Maffei (2014) and Occhipinti et al. (2014) compared variations of GMF polarity in the Tertiary and Cretaceous periods with periods of diversion of families and orders of Angiosperms, and noted that times of polarity reversals coincided with particular steps of plant diversion. They concluded that the sharp decrease of the GMF intensity that coexists with those reversals influenced plant evolution, which implicitly suggests some influence of the GMF on plant physiology (Maffei, 2014; Occhipinti et al., 2014). da Silva and Dobránszki (2016) also implicitly suggested an action of the GMF in plant physiology. Based on multiple observations of modifications of various cell stress markers under diverse MF exposures, they proposed that the MF studied act through the abiotic stress they cause to plants, i.e., via an alteration of the natural magnetic environment. However, the authors did not propose a specific role for the GMF. By reviewing effects of near-null MF in plants and animals, Binhi and Prato (2017) implicitly evoked some action of the GMF on living organisms. Noting the high heterogeneity of the reported MF effects, they proposed some non-specific magnetoreception that would manifest itself as mostly random reaction and that would be based on the interaction of the GMF with the magnetic moments of macromolecules and proteins.

## Cryptochromes as magnetosensors

Diverse mechanisms have been suggested to mediate MF effects in plants. Currently, no evidence supports a mechanism based on the magnetic moment, either of molecules, or of magnetized particles in plants in the microtesla range (see Hore and Mouritsen, 2016). In the millitesla range, however, there is evidence for a so called “normal” MF effect (MFE) on the outcome of various radical pair (RP) reactions such as, for example, in the photosynthesis reaction center (Liu et al., 2005). In contrast, in the microtesla range, a “low field” effect (LFE) has been observed in some experimental models of RP reactions. This is opposite to and weaker than the MFE (see section The magnetic field effect on Cry). In organic molecules, such LFE could so far only be proved in Cry proteins (Maeda et al., 2012).

The Cry proteins belong to the photolyases/Cry flavin adenine dinucleotide (FAD) flavoprotein family. In plants, they mediate the responses to UVA and blue light. Cry have been proved sensitive to MF in the submillitesta range (Maeda et al., 2012). Some of their main functions, such as growth inhibition, seedling de-etiolation or flowering initiation were reported to respond to weak MF and near-null MF (review in Maffei, 2014; da Silva and Dobránszki, 2016; Binhi and Prato, 2017). To date, they are thus the most probable candidates for the mediation of MF effects in the microtesla range, in particular in the Earth strength range (25–65 μT depending on the latitude) (review in Chaves et al., 2011; Ahmad, 2016; Hore and Mouritsen, 2016).

Further supporting the proposal that plant magnetosensitivity could fulfill some function (see above), is the fact that several particular, if not exceptional, physicochemical conditions need to be all satisfied for Cry to be sensitive to MF in the submillitesla range (see Hore and Mouritsen, 2016). Such complexity is unlikely to have evolved without natural selection. Thus, from that point of view also, it seems unlikely that magnetosensitivity of plant Cry has no functional role.

Due to the mechanism of action of a MF on the Cry protein (see section The magnetic field effect on Cry), time-varying MF are not considered here. Diverse effects have been reported in plants under exposure to time-varying, narrow band, MF, mainly extremely low frequencies of electricity (50/60 Hz), and radiofrequencies of wireless communication systems (between about 1 GHz and 3 GHz) (see Maffei, 2014; da Silva and Dobránszki, 2016; Vian et al., 2016). Time-varying MF however could in principle affect Cry only if they are broadband, with frequencies matching the energy level-splitting of electrons in the GMF and the MF from surrounding nuclei, i.e., between about 1 kHz and 100 MHz (Schwarze et al., 2016; Hiscock et al., 2017). At 1 GHz and beyond, it is mainly the electric component of the electromagnetic field, not the magnetic one, that is responsible for the interactions with the living matter (IARC, 2013).

The magnetic sensitivity of Cry is mainly of isotropic nature, meaning that Cry will be affected by a MF whichever its orientation relative to the molecule. The smaller, anisotropic (directional) part of the sensitivity is presumed to provide the magnetic compass-sense in animals (birds in particular) (see Hore and Mouritsen, 2016). In plants, there is no apparent reason to suppose it plays a role. Moreover, one can neglect the anisotropic part in plants also for the following reasons. First, even in the unlikely event that Cry molecules were fixed in cells, the averaging of the anisotropic effect over the entire population of Cry proteins would be equal to zero. Secondly, light polarization—this can elicit an anisotropic response—would only select a part of the population of the Cry molecules, and its effect would be disrupted by light scattering in tissues. Last but not least, the part of the MF effect attributable to anisotropic sensitivity is small compared to that due to the isotropic one (see Hore and Mouritsen, 2016). Thus, in all discussions below, only the intensity of the MF, irrespective of its orientation, is considered.

## The magnetic field effect on cry

**Figure 1:**
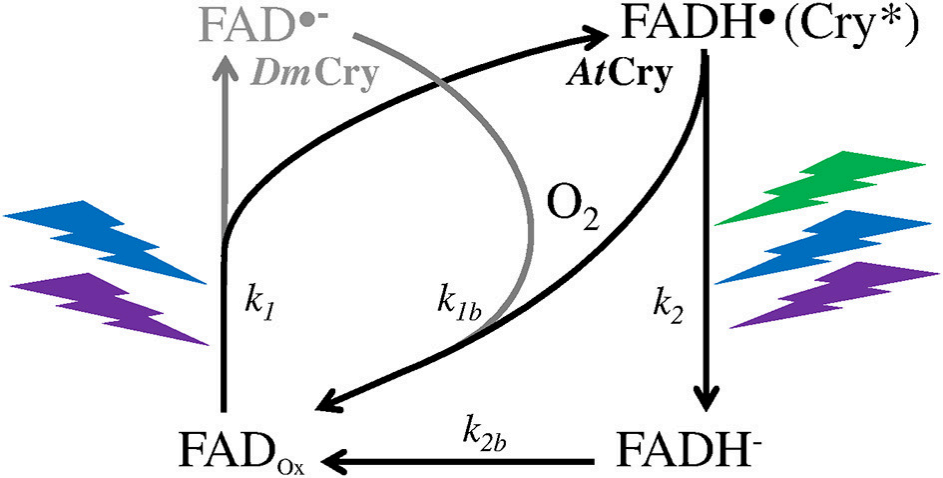
Photocycle of the FAD cofactor of the Cry protein of the plant *Arabidopsis thaliana* (*At*Cry, in black) (Procopio et al., 2016) and of the fruit fly *Drosophila* (*Dm*Cry, in gray) (Arthaut et al., 2017). Under exposure to light (350–500 nm, UVA to blue), the oxidized form of *At*Cry is readily reduced. The radical form (neutral in *At*Cry, anionic in *Dm*Cry) that results from this step is the active one (Cry^*^). The binding of that form with signaling partners causes its phosphorylation and subsequent proteolysis. In *Dm*Cry, FAD^•−^ undergoes direct reoxidation with dioxygen. In *At*Cry, FADH^•^ is either directly reoxidized or fully reduced by light (350–580 nm, UVA to green) and further reoxidized.

**Figure 2:**
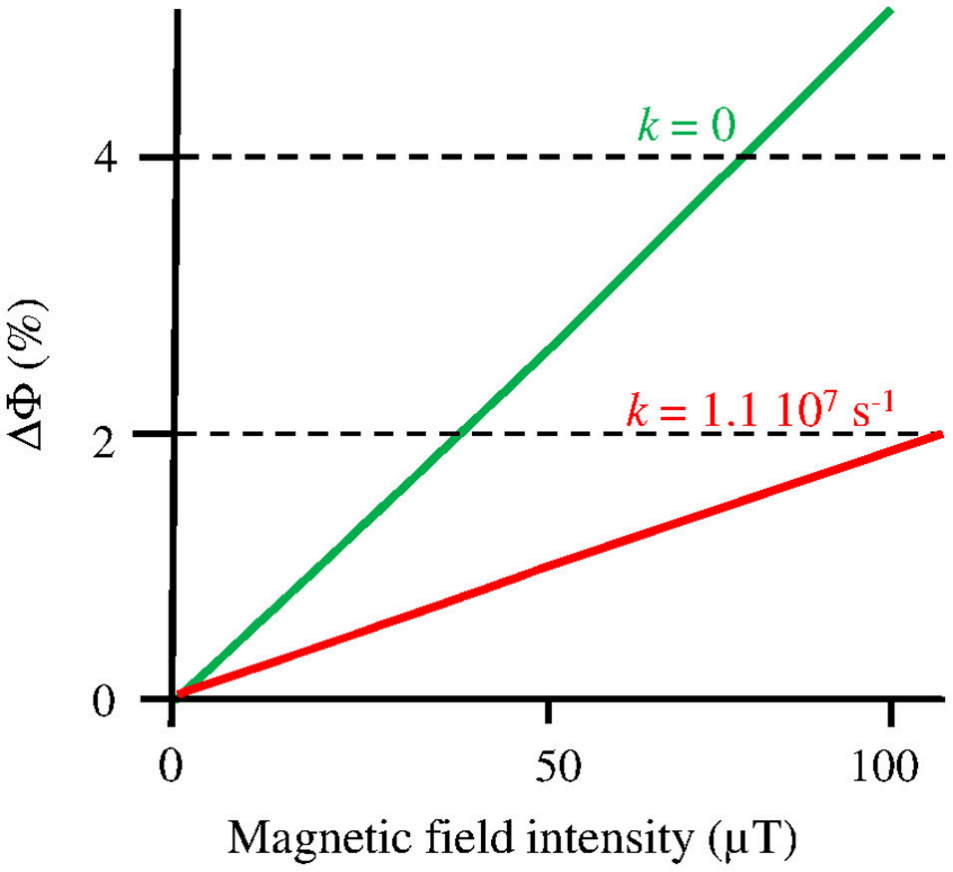
Change (%) of the yield (Φ) of FADH^•^ in *At*Cry 1, thus of the constant *k*_1_ of its photoreduction, according to the intensity *B* of a static MF under flash photolysis (adapted with permission from Maeda et al., 2012, © National Academy of Sciences of the United States of America). The graph focuses on the Earth strength range, where the MF effect is to increase Φ (*k*_1_). The function ΔΦ(*B*) is shown here for *k*_STD_ (*k*) = 1.1 10^7^ s^−1^, where it best fits the experimental observations, and for *k*_STD_ = 0, which can be the case *in vivo* (see text).

A MF acts on plant Cry by influencing an RP reaction of which the outcome is spin-sensitive, i.e., that differs according to the spin state (singlet *vs* triplet) of the RP (see Hore and Mouritsen, 2016). The magnetosensitive RP reaction occurs at the step of the photoreduction of the ground, oxidized state of the FAD cofactor (FAD_ox_) (cf. Figure 1). The RP results from a light-activated electron transfer to FAD from a neighboring tryptophan (Trp) residue of the protein or, possibly, from another donor (see Ahmad, 2016). In contrast to the MFE, that consists in a decrease of the triplet-yield of the RP reaction, the LFE results in an increase of that yield. In an *in vitro* experiment (flash photolysis) on *At*Cry 1, Maeda et al. (2012) have shown that a weak MF affects the yield Φ of the active form or signaling state of Cry (FADH^•^, from here on denoted as Cry^*^) in a way that depends on its intensity (*B*, μT) (Figure 2). The MF effect consists in a relative change of Φ (ΔΦ in %), thus of the constant *k*_1_(Δ*k*_1_) of photoreduction of FAD_ox_ (cf. Figure 1). Indeed, *k*_1_ is directly related to Φ according to

(1) $$k1\;=\sum_{i\;=\;350}^{500}\sigma i\;Ii\;\sim \;\sum_{i\;=\;350}^{500}\varepsilon i\;\Phi \;\text{I}i\;$$

where σ is the photoconversion cross section (m^2^ μ mol^−1^), ε (mol^−1^ m^−1^) is the absorbance, *I* is the light intensity, expressed as the photon fluence rate (μmol m^−2^ s^−1^) and i is the ith wavelength (nm) in the range 350–500 nm (UVA to blue), i.e., the wavelength range that is absorbed by FAD_ox_ (Procopio et al., 2016). As the LFE consists in an increase of *k*_1_, it results in an increase of [Cry^*^]. If magnetic sensitivity fulfills some function, one can assume that, *in vivo*, the LFE will be optimized through protein-protein interactions and a concomitant decrease of the singlet-triplet dephasing rate (*k*_STD_) in the RP (Maeda et al., 2012; Sheppard et al., 2017). For *k*_STD_ = 0, Maeda et al. evaluated the yield change ΔΦ(*B*), thus Δ*k*_1_(*B*), to be of about 2.5% at 50 μT relative to its value at 0 μT (cf. Figure 2).

To evaluate ΔΦ in real life conditions, that is, under continuous illumination, Kattnig et al. (2016) studied the MF effect under repetitive light flashes. In that way, they observed an increase of ΔΦ(*B*) in an experimental flavin-Trp RP model. Under repetitive flashes at an intensity of 0.7 μW/μm^2^ (470 nm), corresponding to about 3 μmol m^−2^ s^−1^, they evaluated that the LFE (Φ increase) that was observed under a single light flash (see Maeda et al., 2012) is multiplied by a factor of up to 5.6. Kattnig et al. (2016) confirmed such an amplification effect in *At*Cry 1. That effect has been attributed to the kinetics of the reoxidation of FADH^•^ (Cry^*^). Indeed, the absolute magnitude of ΔΦ(*B*) increases (or decreases) when the reduction rate of the partner of a flavin-containing RP is faster (or slower) than the one of the flavin reoxidation (cf. Figure 1) – this would be due to a decreased (or increased) reverse electron transfer in the RP (Kattnig et al., 2016).

## Light intensity-dependence of the MF effect on plants

Following Xu et al. (2012), we consider here that any MF effect on plant physiology would be mediated by the impact of that MF on the Cry signaling state ([Cry^*^]). Here we address the dependence on light intensity of that impact and, further, of the effect of Δ[Cry^*^] on Cry-dependent phenotypes.

### *I*-dependence of the MF effect on cry

#### *I*-dependence of the MF effect on the constant *k*_*1*_

The amplification of the MF effect on Φ that Kattnig et al. (2016) observed (see section The magnetic field effect on Cry) appears inversely related to *I*. They evaluated the amplification under continuous illumination to be larger when *I* was lower, rising up to 24 when *I* dropped from about 3 μmol m^−2^ s^−1^ to about 0.5 μmol m^−2^ s^−1^. Such inverse dependence on *I* could be due to the decrease of the rate constant *k*_2_ with decreasing *I*, but would then only exist if the photocycle of Cry is a three-state one like in *At*Cry (cf. Figure 1). Note that, whichever the photocycle (two-state or three-state), temperature (*T*) also affects the reverse redox reaction of both RP partners. *T* might thus also affect the magnitude of that amplification (yet in an unpredictable way) in case the *T*-dependences of the reoxidation of FADH^•^ and of its RP-partner differ. Thus, from the observations and evaluations by Maeda et al. (2012) and Kattnig et al. (2016), the *k*_1_ increase in the GMF (~50 μT) under continuous illumination would be between about 1% and ≥50% compared to zero MF, depending on *k*_STD_ *in vivo* and on light intensity, the increase being larger when these two parameters are lower.

#### *I*-dependence of the MF effect on cry signaling state

For a given change of *k*_1_, the [Cry^*^] change (in %, relative to its value at zero MF), i.e., Δ[Cry^*^](Δ*k*_1_), depends on the kinetics of the whole photocycle of Cry, thus on the rate constants of its respective (first-order kinetics) reactions (cf. Figure 1). These constants themselves depend on the following physical parameters. First, *I* affects the constants *k1* and *k2* (s^−1^) of the photochemical reactions according to

(2) k=∑σi Ii

where i is the ith wavelength (nm) in the range 350–500 nm for *k*_1_, and 350–580 nm (UVA to green) for *k*_2_ (Procopio et al., 2016). Secondly, *T* (in °C) affects the constants *k1b* and *k2b* of the biochemical reactions according to the Arrhenius law, *k* being thus proportional to e^−Ea/RT^, where Ea is the activation energy, R is the universal gas constant, and T is the temperature in kelvin. For many biochemical reactions, the function *k*(T) remains roughly linear between 273 K (0°C) and 293 K (20°C) and becomes exponential only beyond that last temperature. This is notably the case for the rate of reoxidation of the active form of phytochrome B (PhyB) (Legris et al., 2016). Phy is the plant photoreceptor of red and far-red light, and the *T*-dependence of its photocycle is similar to that of Cry 1 (at least when *I* < 100 μmol m^−2^ s^−1^) (Legris et al., 2016). At 0°C and below, most biochemical reactions are blocked. In the Cry photocycle, this should also be the case for the photochemical reactions (Herbel et al., 2013). As a consequence, we consider here the *T*-range between >0° and ≤ 20°C.

In *At*Cry 1 and *At*Cry 2, the constant *k*_2*b*_ is always higher than *k*_2_(see Procopio et al., 2016 and references therein). As a consequence, the reoxidation of FADH^−^ is not a limiting step and the evaluation of [Cry^*^] according to *I* and *T* can be made on basis of the following two-state model

(3) $$A\;\underset{k_{b}}{\overset{k_{a}}{⇄}}\;B$$

where A = FAD_ox_, B = FADH^•^ (Cry^*^), *k*_*a*_ = *k*_1_, and *k*_*b*_ = *k*_2_ + *k*_1*b*_. At the equilibrium, as under continuous illumination, [B] = [B]_eq_ which is itself given by

(4) $${\left[B\right]}_{\text{eq}}\;=\;k_{a}/k_{b}\;{\left[\text{A}\right]}_{\text{eq}}$$

Since the total concentration of A and B together must be constant, [A]_eq_ + [B]_eq_ = [A]_o_ ([A]_o_ is [A] in the absence of light, when [B] = 0). Equation (4) then gives

(5) $${\left[\text{B}\right]}_{\text{eq}}\;=\;\frac{k_{a}/k_{b}}{1+k_{a}/k_{b}}\;{\left[\text{A}\right]}_{\text{o}}$$

From that model, the relative change of [B]_eq_ (Δ[B]_eq_) that is caused by a given change of *k*_*a*_ expressed as *x* = log (*k*_*a*_/*k*_*b*_) – *k*_*b*_is constant here –, at given values of *I* and *T*, corresponds to an inverted sigmoid function centered at *x* = −0.079, with a first derivative equal to −0.585, and that is thus defined as follow

(6) $$f\left(x\right)=\frac{1}{1+e^{2.31(x+0.079)}}$$

where ***f*** (x) gives the solution for Δ[B]_eq_/Δ*k*_*a*_ (Δ[Cry^*^]/Δ*k*_1_) according to log (*k*_*a*_/*k*_*b*_) for the case where Δ*k*_*a*_ (Δ*k*_1_) = 20%, that is within the range of values possibly caused by the GMF, i.e., 1–50% (Maeda et al., 2012; Kattnig et al., 2016). Note ***f***(x) remains similar within that range. For Δ*k*_*a*_ = 1 or 50%, it is, respectively, slightly shifted to the right (centered at x ~0) or to the left (centered at x = −0.5), and its slope remains similar. Δ[Cry^*^]/Δ*k*_1_ is then calculated for different *I* and *T* values, with *x* = log (*k*_1_/*k*_2_ + *k*_1*b*_) at each respective values. Calculations are made for *I* summed over the range 350–500 nm (UVA to blue), based on the following parameters:

- σ_1_ = 2 10^−5^ m^2^/μmol, according to *in vivo* evaluation (Procopio et al., 2016), and with extrapolating σ at 450 nm to the whole range 350–500 nm (only small consecutive error)
- σ_2_ = 2 10^−6^ m^2^/μmol, according to an σ_1_/σ_2_ ratio of about 15 in Cry 2 (Procopio et al., 2016) and with multiplying σ_2_ by 1.5 in order to take into account the fact that FADH^•^ absorbs light from UVA down to the green range (*I*_350−580nm_ / *I*_350−500nm_ ~ 1.5 for the solar spectrum on Earth)
- *k*_1*b*_ = 10^−4^ *T* in the *T*-range considered, in accordance with a τ_1/2_ of about 6 min (thus *k*_1*b*_ = 0.002 s^−1^) at 20°C in the dark for FADH^•^ in *At*Cry 1 (Herbel et al., 2013).

**Figure 3:**
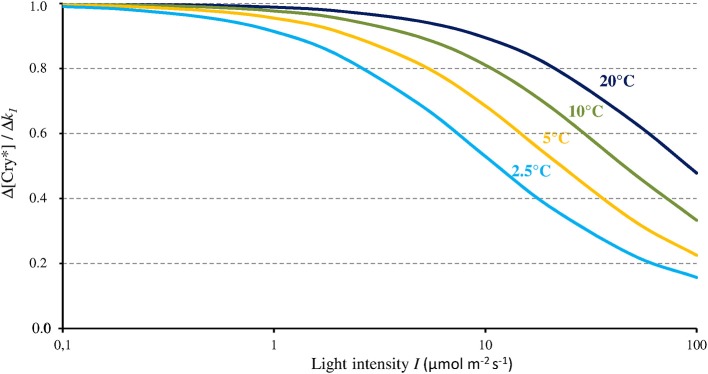
Change of the concentration of the active form of Cry (Cry^*^) relative to the change of *k*_1_ (as caused by a MF) as a function of blue light intensity (*I*, 350–500 nm) and for different temperatures. From that relationship, a MF can thus only express its full effect at low light intensities.

As Figure 3 shows, the magnitude of the effect of any given change of *k*_1_ on [Cry^*^] depends on *I* and *T*. In accordance with its dependence on the ratio *k*_1_ / (*k*_2_ + *k*_1*b*_), the magnitude of the effect of Δ*k*_1_ on [Cry^*^] (each expressed in % of change) is maximal (Δ[Cry^*^] = Δ*k*_1_) below light intensities of about 1 μmol m^−2^ s^−1^, and it decreases in a *T*-dependent way at higher intensities and vanishes beyond 100 μmol m^−2^ s^−1^. Modifying σ_1_ and/or σ_2_ within a range of plausible values (see Procopio et al., 2016 and references therein) does not change the shape of the function shown on Figure 3 but slightly shifts it to the right or the left.

The present evaluation and the one by Kattnig et al. (2016, see section The magnetic field effect on Cry) converge toward a 1/*I*-dependence of the MF effect on Cry under continuous illumination. From the 1/*I*-dependence of its relative magnitude, that effect fully expresses itself only below 1–10 μmol m^−2^ s^−1^ of intensity. And from the 1/*I*-dependence of its absolute magnitude under continuous illumination, that effect could be further amplified below these light intensities. Such inverse dependence on light intensity let suppose *At*Cry 2 to be also subject to the influence of static MF. Indeed, in contrast to *At*Cry 1 that is light stable, the reduced form of *At*Cry 2 is promptly degraded under illumination and its function is presumed effective only under low light intensity, where a MF effect can precisely occur.

### *I*-dependence of consecutive phenotypic changes

By affecting *k*_1_, thus [Cry^*^], a MF possibly affects the function of Cry. Of the various responses reported to weak static MF in plants (review in Maffei, 2014; da Silva and Dobránszki, 2016; Binhi and Prato, 2017), several were Cry-dependent. It is relevant to review them again in the light of the present developments.

#### MF effects reported on cry-dependent phenotypes

Here we only consider studies published either before 2,000 and reproduced since or published after 2,000 in peer-reviewed journals written in English language, and that addressed effects on Cry-dependent phenotypes with also mentioning the light intensity used. As Table 1 shows, the studies did all report MF effects under low intensities of light (≤63 μmol m^−2^ s^−1^ of white light, ≤12 μmol m^−2^ s^−1^ of blue light). In contrast, results obtained under higher light intensities were contradictory. Thus, the MF effects reported in plants suggest that a MF can affect Cry-dependent phenotypes under low light intensities. As for the nature of reported effects, most are in line with what can be predicted from the LFE on *k*_1_. Indeed, effects reported under zero MF for example were mainly growth increase and/or delayed or reduced flowering, which is consistent with a decrease of [Cry^*^] at zero MF compared to 30–50 μT (most studies used the local GMF as control).

**Table 1:** Effects of static MF or zero MF (<2 μT) reported in plants that can be related to Cry function, and that have been published in peer-reviewed journals written in English since the year 2,000 (λ = wavelength, WT = wild-type). All studies have been performed at about 21°C (25°C in Rakosy-Tican et al., 2005).

**Plant species**	***B* (μT)**		**Light exposure**		**Results**	**References**
	**Test**	**Control**	**λ(nm)**	***I*(μmol m^−2^ s^−1^)**		
*Solanum spp*.	0	47	white	25^a^	↗ hypocotyl growth (stem length), statistically significant or not	Rakosy-Tican et al., 2005
*Arabidopsis t*.	500	33/44	465/633	13/46/80	↘ hypocotyl growth in WT (not in *Cry 1,2 –/–*, or under red light)	Ahmad et al., 2007
*Arabidopsis t*.	500	50	470	80^b^	No change in hypocotyl growth	Harris et al., 2009
	50/10^3^	0			No change in hypocotyl growth	
	10^5^	0		80, 50^b^	No change in hypocotyl growth	
	10^5^	0		12^b^	↘ hypocotyl growth	
*Arabidopsis t*.	0	45	white	33	↗ hypocotyl growth, delayed flowering, changes of expression of Cry-signaling related genes	Xu et al., 2012
*Arabidopsis t*.	0	45	white	35	↘ biomass accumulation at 35 days (due to delayed flowering) ↘ harvest index (−20%)	Xu et al., 2013
*Arabidopsis t*.	0/500	45	460	10	↘ phosphorylation of Cry 2, ↗ dephosphoryl. of Cry 1, 2 (0 μT) ↗ phosphoryl. of Cry 1, 2 (500 μT)	Xu et al., 2014
*Lemna Minor*	4/100	30	white	63	↗ growth rate (4 μT) ↘ growth rate (not signif.) (100 μT)	Jan et al., 2015
*Arabidopsis t*.	0	45	460/650	10	↘ flowering under blue light in WT in 6 h/6 h Light/Dark cycles (not in *Cry 1,2 –/–* or under red light)	Xu et al., 2015
*Arabidopsis t*.	0	45	460	10	↘ gibberelins and flowering-related genes in WT (not in *Cry 1,2 –/–*)	Xu et al., 2017

a *2,000 lux from cool white fluorescent lamp*.

b *From intensities in W/m^2^ (1 W/m^2^ ~ 4 μmol m^−2^ s^−1^)*.

#### Hypocotyl growth as an exemplary phenotype

**Figure 4:**
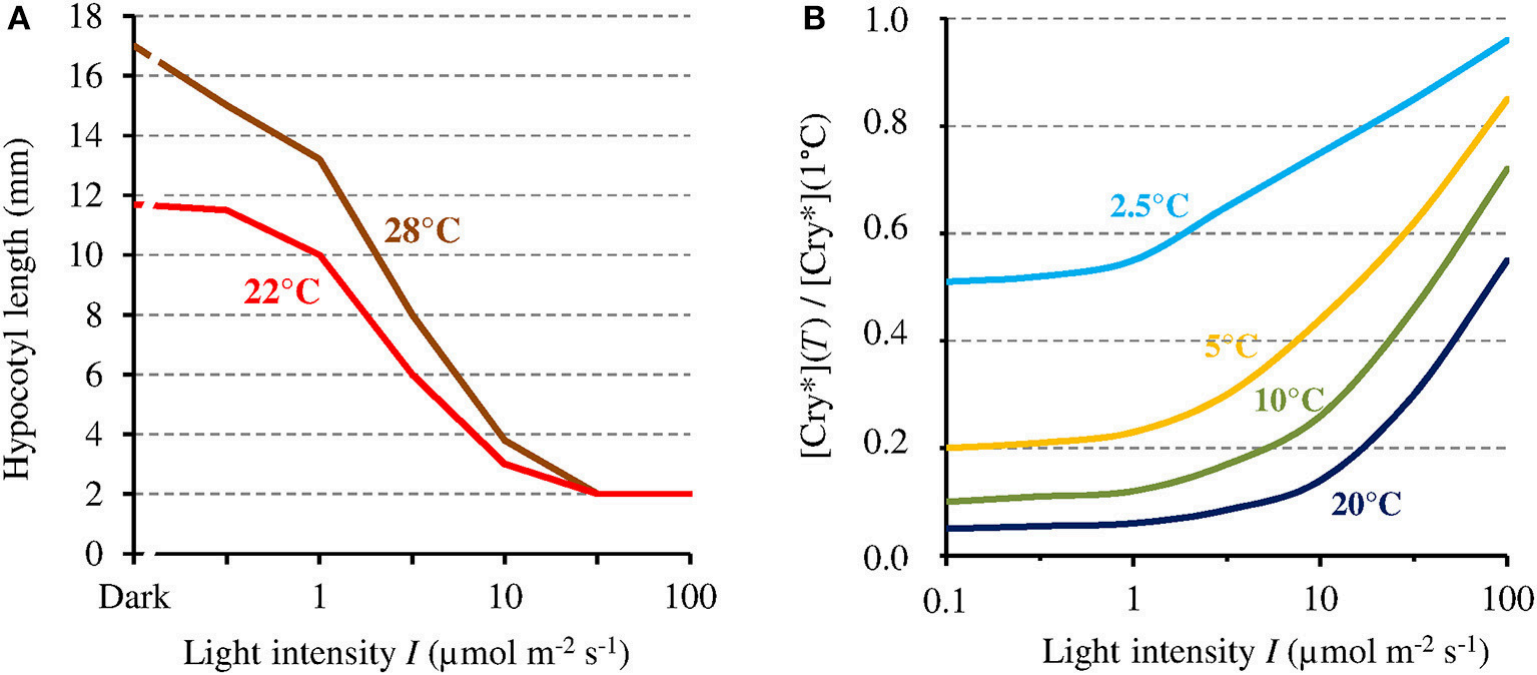
**(A)** Hypocotyl growth in *At*Cry 1 according to blue light intensity (*I*), respectively at 22° and 28°C (adapted with permission from Ma et al., 2016, © National Academy of Sciences of the United States of America). **(B)** [Cry^*^] expressed as its value relative to that at 1°C, at different temperatures and as a function of *I*, as evaluated from the present model (this assumes the function *k*_1*b*_(*T*) to be linear and is thus only valid at ≤20°C [see text]). [Cry^*^] varies with *I* and *T* in a way comparable but inverse to hypocotyl growth.

In view of the 1/*I*-dependence of the MF effect on [Cry^*^], that effect could in principle only affect related phenotypes that are likewise 1/*I*-dependent. Hypocotyl growth has been one of the most studied phenotype in plants. Noteworthy here, like the MF effect on [Cry^*^], hypocotyl growth varies inversely with *I*. For that particular phenotype, indeed, the absolute change that is caused by a given relative change of [Cry^*^] is the largest when *I* is the lowest. This is illustrated by the dependence on blue light intensity of the effects of temperature on *At*Cry 1-dependent hypocotyl growth (Ma et al., 2016; cf. Figure 4A). Figure 4B shows the dependence of [Cry^*^] on *T*, expressed as the ratio [Cry^*^](*T*) / [Cry^*^](1°C) according to *I*, as evaluated from our two-state model. Figure 4B deserves comparison with the results of Ma et al. (2016) (Figure 4A). Indeed, like the *T*-dependence of growth reported by these authors, the one of [Cry^*^] evaluated here is mostly marked between 1 μmol m^−2^ s^−1^ and ~100 μmol m^−2^ s^−1^. Further illustrating the fact that a given Δ[Cry^*^] causes an effect that varies as 1/*I*, the impact of a given *T* change on growth is the largest at <1 μmol m^−2^ s^−1^ (Figure 4A) even though the variation of [Cry^*^] with *T* is then the lowest (at least for *T* ≥ 10°C) (Figure 4B). And the comparison between Figures 4A,4B supports the view that the effects of temperature on Cry-dependent growth are partly mediated by the photocycle, as is otherwise the case for Phy-dependent growth (Legris et al., 2016). Besides of the regulation of hypocotyl growth, other functions of Cry are low light-dependent as well, such as for example, the inhibition of root growth (Zeng et al., 2010).

## Possible relevance of cry magnetosensitivity to plant ecology

From the present developments, Cry magnetosensitivity in plants would only express itself in low light conditions, with the MF affecting the signaling state of Cry in a way possibly similar to temperature. Now, the actual magnitude of the magnetic sensitivity of Cry, i.e., Δ*k*_1_(*B*), *in vivo* is not known to date. Yet, in case it is large enough for the GMF to significantly affect [Cry^*^], as published MF effects in plants suggest, then the magnetic sensitivity of Cry could take part to the adaptation of its signaling state to low light conditions, such as they prevail under the canopy and/or at high latitudes (depending on seasons).

### Under the canopy

**Figure 5:**
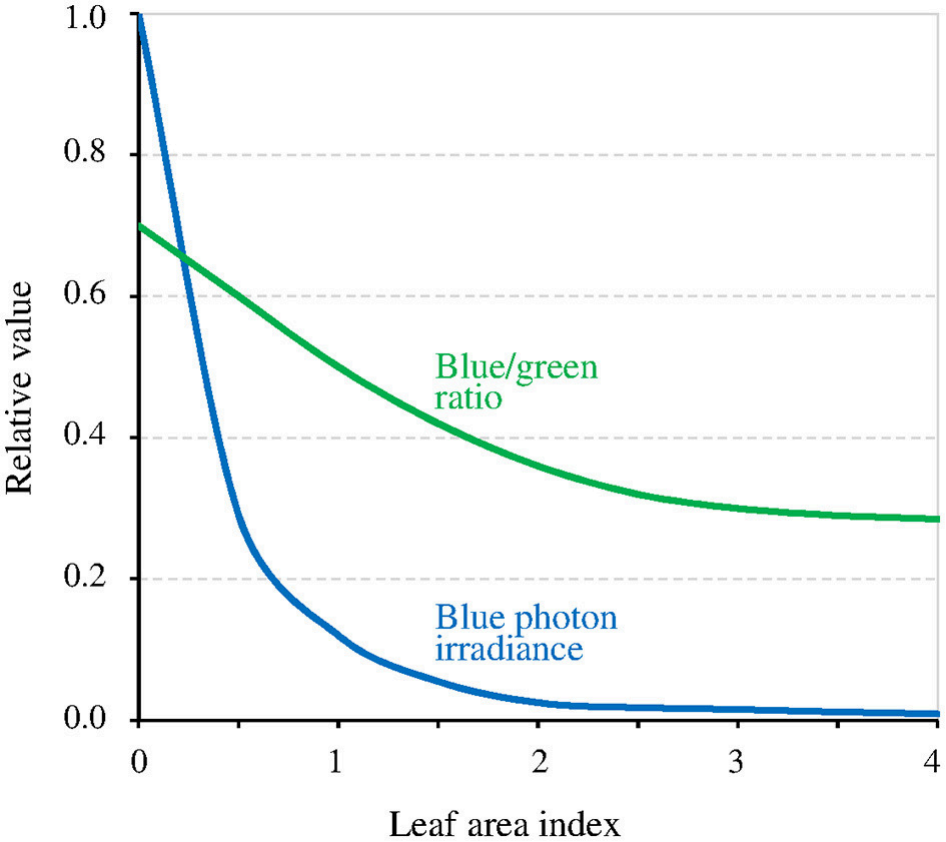
Relative value of blue light intensity and of the blue/green ratio as a function of the density of the canopy coverage expressed as the leaf area index (an index of 1 corresponds to a single layer of leaves) (adapted with permission from Sellaro et al., 2010, © American Society of Plant Biologists).

The canopy coverage dramatically reduces *I* (−90% for a single layer of leaves, thus much lower for a dense canopy; Figure 5). The consequence is that it can reveal or enhance the effect of the GMF, this last then possibly also contributing to shade avoidance in plants (see Fraser et al., 2016). Furthermore, the light intensity below which the MF effect arises is related to *T* (Figure 3). Thus, at lower latitudes, due to higher temperatures there, the canopy effect can already arise despite *B* being lower there. The canopy also favors the MF-effect by the way it filters light. Indeed, leaves absorb blue light more than green light, thereby decreasing the blue/green ratio (−50%) (Sellaro et al., 2010; Figure 5). The consequence is a decrease of the σ_1_/σ_2_ ratio, thus of the ratio *k*_1_ / (*k*_2_ + *k*_1*b*_), and a concomitant shift toward the right (towards higher light intensities) of the function Δ[Cry^*^](Δ*k*_1_).

### At high latitudes

**Figure 6:**
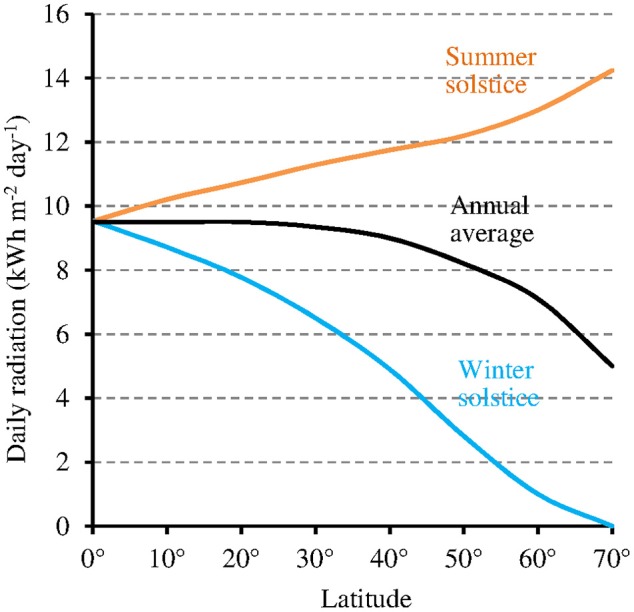
Relation between latitude and total daily radiation under clear sky (annual average and at the two solstices of the year) (source: pvlighthouse, http://pveducation.org/pvcdrom/calculation-of-solar-insolation).

**Figure 7:**
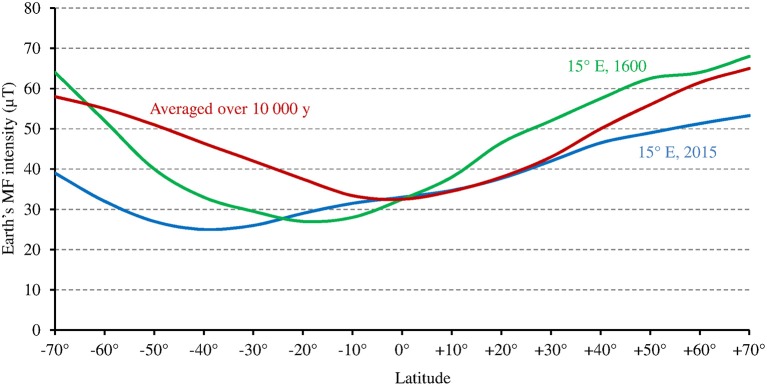
Relation between Earth's MF intensity and latitude, averaged over the last 10,000 years according to the models CALS10k.2 or HFM.OL1.A1 (standard deviation is 2–5 μT) (adapted with permission from Constable et al., 2016, © Elsevier B.V.), and at 15° longitude E, where the studies by Stenøien et al. (2002) and Ranade and García-Gil (2013) (see text) were performed, respectively in the year 1600 (source: World data center for geomagnetism, Kyoto, http://wdc.kugi.kyoto-u.ac.jp/), and in 2015 (source: World Magnetic Model 2015, https://www.ngdc.noaa.gov/geomag/WMM/data/WMM2015/WMM2015_F_MERC.pdf).

While at low latitudes, *I* during the light phase is in the average ≥500 μmol m^−2^ s^−1^ in the UVA-blue range (−50% in case of overcast sky), it can be up to tenfold lower at high latitudes (50 μmol m^−2^ s^−1^ in the average at 60° of latitude) in winter ^1^ (see also Figure 6 where light intensity is expressed as the total radiation received per day and per year). As a consequence, the magnitude of the MF effect on Cry will vary depending on time and place on Earth: it will be close to zero at low latitude during most of the day, and close to its maximum at high latitude in cold and intermediate seasons, when light levels are low. In addition, the MF intensity gradient that currently exists in the northern hemisphere, between the equator (*B* close to 30 μT) and the pole (*B* close to 60 μT) further amplifies the latitudinal variation of the effect of the GMF on Cry. In the southern hemisphere, the current situation differs from that in the northern one (Figure 7). However, this seems to be an exception to the average situation in the last 10,000 years (Constable et al., 2016; Figure 7) and may be more. Of note, since millions of years, in spite of the many reversals of its polarity, the GMF has been dipolar in the average, with an axis that roughly coincided with that of Earth rotation (Driscoll, 2016).

Noteworthy for the relationship between the Earth's MF effect and latitude are studies of latitudinal clines in the sensitivity of hypocotyl growth to light in Scott pine (Ranade and García-Gil, 2013) and in *Arabidopsis* (Stenøien et al., 2002) at high latitudes (between 58° and 68°N at about 15° of longitude E, see corresponding GMF intensity on Figure 7). In both plants, such cline—this reflects genetic or epigenetic adaptation—could be observed for the sensitivity to red and/or far red light, but not to blue light (this remained stable across latitudes). Thus, one might consider that at high latitudes, temperature alone (as in PhyB) cannot cause changes to the photocycle that are large enough to allow the photoreceptor function to adapt. Hence, some genetic/epigenetic change is required. But this could not be the case for the sensitivity to blue light because the magnetic sensitivity of Cry would compensate for this.

## The case of animal cry

In animals, Cry proteins are currently the most likely magnetosensor candidates (see Hore and Mouritsen, 2016). Hence, it seems relevant to address the question of the light intensity-dependence in animal Cry also.

### Type I cry of insects

Type I Cry of the fruit fly *Drosophila* (*Dm*Cry) and other insects are responsible for the light-entrainment of circadian biorhythms like in plants, but they are also presumed to mediate magnetoreception of insects. Indeed, diverse Cry-dependent responses to weak MF have been reported in *Drosophila* (review in Sheppard et al., 2017). In contrast to *At*Cry however, a MFE, but no LFE, has been shown in *Dm*Cry *in vitro* (Sheppard et al., 2017). The explanation for the absence of a LFE *in vitro* could reside in structural difference between *At*Cry and *Dm*Cry (Nohr et al., 2016), and/or in the possible existence of some associated mechanism, such as radical scavengers, that would amplify the MF effect in *Drosophila in vivo* (Kattnig and Hore, 2017). As the photocycle of *Dm*Cry is a two-state one (cf. Figure 1), and considering its parameters (Arthaut et al., 2017), the MF effect must vary as 1/*I* like in *At*Cry. However, the photoconversion cross section (σ) of FAD_ox_ is one order of magnitude larger than σ_1_ in *At*Cry (the constant of reoxidation is comparable) (Arthaut et al., 2017). As a consequence, the ratio *k*_*a*_/*k*_*b*_ (cf. Equation 2) is larger in *Dm*Cry than in *At*Cry, and the transition between the maximum and the minimum of the function Δ[Cry^*^](Δ*k*_1_) (Figure 3) is shifted toward lower light intensities. If a LFE is proved in *Dm*Cry, then one can consider the possibilities that (a) in insects, as in plants, weak MF affect the expression of the clock proteins that vary inversely with *I* and that (b) the magnetic compass-sense of insects is 1/*I*-dependent.

### Type II cry of vertebrates

Like type I Cry, type II Cry of vertebrates are involved in the regulation of circadian biorhythms. However, that role in vertebrates is currently considered independent of light in any instance, even in cells and tissues that are exposed to light (retina and skin) (see Michael et al., 2017). As light is required for the formation of the magnetosensitive RP in Cry, the present developments cannot apply to that particular role of type II Cry of vertebrates. One possible exception could however be the clock function of certain Cry of the retina. Indeed, in mammals, some data suggest a photocycle to exist for Cry (Cry 2 in humans) involved in the circadian clock function in the retina (reviews in Michael et al., 2017; Vanderstraeten, 2017). And in humans, it has been suggested that Cry of the retina mediate health effects of extremely low frequency MF of electricity (see Vanderstraeten et al., 2015) and MF effects on human visual acuity (Thoss and Bartsch, 2017). These two hypotheses thus suppose retinal Cry to have some light-dependent function. Interestingly, the existence of a photocycle for Cry in mammal retina could also help explaining the still imperfectly understood increase of the concentration of reactive oxygen species (ROS) that mediate the photochemical damages that are caused by blue light in the retina (Hunter et al., 2012). Indeed ROS production has been associated with Cry reoxidation, both in the context of the three-state photocycle of *At*Cry (El-Esawi et al., 2017) and of the two-state cycle of *Dm*Cry (Arthaut et al., 2017).

With respect to the likely role of type II Cry in the light-dependent magnetic compass sense of birds, experimental observations generally agree with the present evaluation of low light-dependence of the MF effect on Cry. Indeed, the bird compass sense has been shown effective at very low light intensities (e.g., starlight, cf. Hore and Mouritsen, 2016), and observations suggest that it varies as 1/*I*. Using monochromatic light, Wiltschko et al. (2010) observed a 1/*I*-dependence for magnetoreception in birds, with cancelation of the compass-sense when *I* rises from about 0.1 μmol m^−2^ s^−1^ at 424 nm to only 0.5 μmol m^−2^ s^−1^. From these observations, the bird magnetic sense would be effective only before sunset or after sunrise. Such observation suggests that σ_1_ is much larger in Cry of birds than in *At*Cry, and/or that the magnetic sense in birds requires a large chemical amplification of the MF effect, hence a very low light intensity (see section *I*-dependence of the MF effect on the constant *k*_1_).

In general, the effectiveness of avian magnetoreception at very low light intensities supports the view that, like in plant Cry, the magnetosensitive step in type II Cry is the photoreduction of FAD_ox_ (cf. Kattnig and Hore, 2017). As can be seen in Equation 5, the MF will affect the redox balance of Cry only when *k*_*a*_ (the magnetosensitive rate constant) is equal to or lower than *k*_*b*_ (see also Figure 3). In the circumstances of both homeothermy in birds (*T* constant around 40°C) and of low light intensity, that condition will be met only if *k*_*a*_ is the photochemical reaction rate (*k*_*a*_ is then low) and *k*_*b*_, the thermal one (*k*_*b*_ is then high), such as in the case of *At*Cry (see section *I*-dependence of the MF effect on Cry signaling state). Recently, it has been proposed that in birds, the magnetosensitive RP reaction takes places, not at the step of the photoreduction of FAD_ox_, but at the one of the reoxidation of FADH^−^ (Wiltschko et al., 2016) (FAD^•−^ in the context of a two-state photocycle). Whether the Cry photocycle is a three-state one (*k*_*a*_ = *k*_2*b*_) or a two-state one (*k*_*a*_ = *k*_1*b*_) (cf. Figure 1), such proposal seems incompatible with magnetoreception at 40°C and at low light intensity. In these circumstances indeed, *k*_*a*_ will always be (much) higher than *k*_*b*_. And in the context of a three-state cycle, one can then assume that most FADH^•^ will be directly reoxidized (*k*_1*b*_ >> *k*_2_), thus that no or little oxidation of FADH^−^ will occur.

## Research avenues

The MF effects reported so far in plants have been at most of moderate magnitude (review in Maffei, 2014; da Silva and Dobránszki, 2016; Binhi and Prato, 2017). Two comments should be made in this respect. First, all studies that mentioned light intensities have used ≥10 μmol m^−2^ s^−1^ of blue light or ≥25 μmol m^−2^ s^−1^ of white light. Now, as abovementioned, it seems that a MF can express its full effect only at lower light intensities (see section *I*-dependence of the MF effect on Cry). Secondly, the mostly studied endpoints were of morphological nature (growth, flowering…). While these are easier to study, such endpoints are less sensitive and specific than the expression of some particular genes or proteins that are directly regulated by Cry. Indeed, like many aspects of plant physiology, metabolism and development, growth is the result of complex interplay between several key clock proteins of which the respective expression itself depends on various parameters, blue light (thus [Cry^*^]) being only one (see Gardner et al., 2006). This is also reflected by the high variability of hypocotyl growth that can be observed under a given light intensity and temperature in *Arabidopsis* accessions that originate from roughly the same location (see e.g., Maloof et al., 2001). As a consequence, study endpoints of choice would be clock proteins of which the expression varies as 1/*I*, and that are downregulated by Cry^*^. And light intensities of ≤0.1–1 μmol m^−2^ s^−1^ should be preferentially used in order to maximize the MF effect. This could also apply to studies of animal magnetoreception.

## Conclusions

The magnitude of the MF effect on the signaling state of plant Cry appears inversely related to light intensity, the consequence of which being that MF could thus affect phenotypes that also vary inversely with light intensity. Based on reported MF effects in plants, MF intensities could be effective down to the Earth strength range. A role for the magnetosensitivity of plant Cry could thus be the adaptation of its signaling state to low light conditions. Further studies might focus on effects of weak MF on phenotypes that vary inversely with light intensity, but under light intensities ≤1 μmol m^−2^ s^−1^, thus lower than those used so far. Sensitive and specific endpoints are the expression of clock proteins that are downregulated by cryptochromes. The present considerations probably also concern type I Cry of insects, not only with respect to their clock function but also their probable role in magnetoreception.

## Author contributions

JV proposed and developed the concepts. All authors contributed to the redaction of the article.

### Conflict of interest statement

The authors declare that the research was conducted in the absence of any commercial or financial relationships that could be construed as a potential conflict of interest.

## Footnotes

^1^*https://www2.pvlighthouse.com.au/calculators*, parameters used: global power, perpendicular incidence, IEC standard for AM1.5g spectrum
