# Corrigendum: Low-Light Dependence of the Magnetic Field Effect on Cryptochromes: Possible Relevance to Plant Ecology

**DOI:** 10.3389/fpls.2018.01459

**Published:** 2018-09-28

**Authors:** Jacques Vanderstraeten, Philippe Gailly, E. Pascal Malkemper

**Affiliations:** ^1^Environmental and Work Health Research Center, School of Public Health, Université Libre de Bruxelles, Brussels, Belgium; ^2^Institute of Neuroscience, Université Catholique de Louvain, Brussels, Belgium; ^3^Department of General Zoology, Faculty of Biology, University of Duisburg-Essen, Essen, Germany; ^4^Department of Wildlife Management, Faculty of Forestry and Wood Sciences, Czech University of Life Sciences, Praha, Czechia

**Keywords:** *Arabidopsis thaliana*, clock proteins, geomagnetic field, light intensity, magnetoreception, plant growth, static magnetic fields

In the original article, there was an error. The definition of ***f*** (x) (Equation 6) requires additional clarification, particularly the approach used to calculate Δ[Cry^*^]/Δ*k*_*1*_.

A correction has been made to the section LIGHT INTENSITY-DEPENDENCE OF THE MF EFFECT ON PLANTS, subsections I-Dependence of the MF Effect on Cry, and I-Dependence of the MF Effect on Cry Signaling State, Paragraph 3.

“… where ***f*** (x) gives the solution for Δ[B]_eq_/Δ*k*_*a*_ (Δ[Cry^*^]/Δ*k*_1_) according to log (*k*_*a*_/*k*_*b*_) for the case where Δ*k*_*a*_ (Δ*k*_1_) = 20%, that is within the range of values possibly caused by the GMF, i.e., 1–50% (Maeda et al., 2012; Kattnig et al., 2016). Note ***f*** (x) remains similar within that range. For Δ*k*_*a*_ = 1 or 50%, it is, respectively, slightly shifted to the right (centered at x ~ 0) or to the left (centered at x = −0.5), and its slope remains similar. Δ[Cry^*^]/Δ*k*_1_ is then calculated for different *I* and *T* values, with *x* = log (*k*_1_/*k*_2_ + *k*_1*b*_) at each respective values.”

The authors apologize for this error and state that this does not change the scientific conclusions of the article in any way.

The original article has been updated.

## Conflict of interest statement

The authors declare that the research was conducted in the absence of any commercial or financial relationships that could be construed as a potential conflict of interest.
